# 'Sexual addiction': diagnosis and treatment in clinical practice

**DOI:** 10.1186/1753-6561-6-S4-P33

**Published:** 2012-07-09

**Authors:** B Hughes

**Affiliations:** 1 School of Social Work and Social Policy, Trinity College Dublin, Ireland

## Introduction

In western medicine emerging research evidence suggests a distinct psychological/biological phenomenon that manifests itself in 'out of control' sexual behaviour. Historically, this behaviour has been described as hypersexuality and compulsive sexual behaviour. Recently it is popularly called 'sexual addiction'.

The aim of this study is to obtain a clear understanding of the diagnostic and treatment issues experienced by healthcare providers who deal with the concept of ´sexual addiction' in clinical practice.

## Methods

A qualitative approach was used. Data collection includes: pilot-study, focus-groups, questionnaires and interviews involving 87 adult participants consisting of 43 treatment providers who work with this phenomenon in clinical practice and 44 self-identified 'sexual addicts'. Ethical approval was obtained from the schools' ethics committee and consent was given by the participants. Interpretative Phenomenological Analysis (IPA) and Thematic Analysis (TA) are used for data analysis.

## Results

Treatment providers report the presentation of a clinical condition popularly referred to as 'sexual addiction'. The main characteristics of this condition are intense sexual urges, recurring fantasies and out of control sexual behaviour. Treatment providers who clinically deal with this are working in the areas of medicine, sexual health, psychiatry, psychology and psychotherapy. The major clinical challenge is the lack of agreement on criteria and the absence of a scientific classification for this condition. Other concerns include diagnosis, treatment, disclosure and understanding the aetiology of 'sexual addiction'. Additionally, there is a high incidence of dual addiction and co-morbid psychological conditions among those who present with 'sexual addiction'. There is a lack of clarity whether the 'sexually addictive' behaviour is the primary condition or whether it is a symptom of an underlying condition. In addition to a catalogue of negative consequences the 'sexual addict' is vulnerable to contracting sexual disease. Treatment is a multifaceted combination of medication, psychotherapy, 12-step fellowship programmes and education. There is a wide range of opinion among treatment providers on the concept of 'sexual addiction'.

## Conclusion

Empirical research is required to investigate and critique the concept of 'sexual addiction'. Diagnostic criteria and classification need to be determined. Clinical training is necessary to diagnose, assess and treat this behaviour.

